# Metastatic susceptibility locus, an 8p hot-spot for tumour progression disrupted in colorectal liver metastases: 13 candidate genes examined at the DNA, mRNA and protein level

**DOI:** 10.1186/1471-2407-8-187

**Published:** 2008-07-01

**Authors:** Donia P Macartney-Coxson, Kylie A Hood, Hong-jun Shi, Teresa Ward, Anna Wiles, Rosemary O'Connor, David A Hall, Rod A Lea, Janice A Royds, Richard S Stubbs, Serena Rooker

**Affiliations:** 1 Wakefield Gastroenterology Research Institute, Wellington, New Zealand; 2 Institute of Environmental Science and Research, Kenepuru Science Centre, Porirua, New Zealand; 3 Dunedin School of Medicine, University of Otago, Dunedin, New Zealand; 4 Cell Biology Laboratory, Department of Biochemistry, University College Cork, Cork, Ireland; 5 Capital and Coast Health, Wellington Hospital, Wellington, New Zealand

## Abstract

**Background:**

Mortality from colorectal cancer is mainly due to metastatic liver disease. Improved understanding of the molecular events underlying metastasis is crucial for the development of new methods for early detection and treatment of colorectal cancer. Loss of chromosome 8p is frequently seen in colorectal cancer and implicated in later stage disease and metastasis, although a single metastasis suppressor gene has yet to be identified. We therefore examined 8p for genes involved in colorectal cancer progression.

**Methods:**

Loss of heterozygosity analyses were used to map genetic loss in colorectal liver metastases. Candidate genes in the region of loss were investigated in clinical samples from 44 patients, including 6 with matched colon normal, colon tumour and liver metastasis. We investigated gene disruption at the level of DNA, mRNA and protein using a combination of mutation, semi-quantitative real-time PCR, western blotting and immunohistochemical analyses.

**Results:**

We mapped a 2 Mb region of 8p21-22 with loss of heterozygosity in 73% of samples; 8/11 liver metastasis samples had loss which was not present in the corresponding matched primary colon tumour. 13 candidate genes were identified for further analysis. Both up and down-regulation of 8p21-22 gene expression was associated with metastasis. ADAMDEC1 mRNA and protein expression decreased during both tumourigenesis and tumour progression. Increased STC1 and LOXL2 mRNA expression occurred during tumourigenesis. Liver metastases with low DcR1/TNFRSF10C mRNA expression were more likely to present with extrahepatic metastases (p = 0.005). A novel germline truncating mutation of *DR5/TNFRSF10B *was identified, and *DR4/TNFRSF10A *SNP rs4872077 was associated with the development of liver metastases (p = 0.02).

**Conclusion:**

Our data confirm that genes on 8p21-22 are dysregulated during colorectal cancer progression. Interestingly, however, instead of harbouring a single candidate colorectal metastasis suppressor 8p21-22 appears to be a hot-spot for tumour progression, encoding at least 13 genes with a putative role in carcinoma development. Thus, we propose that this region of 8p comprises a metastatic susceptibility locus involved in tumour progression whose disruption increases metastatic potential.

## Background

Mortality from colorectal cancer (CRC), the fourth most frequent cause of cancer deaths, is mainly due to metastatic liver disease. Much is known about the adenoma-carcinoma progression of CRC [1-3] and sporadic CRC is recognised as a heterogeneous and complex disease involving many genes and pathways [4,5]. There has been intensive analysis of the prognostic value of molecular markers for CRC in risk assessment and disease management [6-11]. Despite intense study of the metastatic process many aspects of its molecular genetic basis remain unclear. Improved understanding of the molecular events underlying metastasis is crucial for the development of new methods for early detection and treatment of colorectal cancer.

Traditionally, loss of heterozygosity (LOH) analyses were used to map regions harbouring tumour suppressor genes; this method exploits Knudson's two hit hypothesis of tumorigenesis [12] We reasoned that LOH analyses could be used to map chromosomal regions specifically disrupted in metastases, and might therefore highlight the presence of a gene(s) involved in metastasis.

Chromosome 8p is frequently lost in CRC, many studies implicate loss in later stage disease and metastases [13-15], and several 8p genes have been implicated in metastasis [16-19]. However, to date no strong candidate CRC metastasis suppressor has been identified showing loss of expression and/or function in a significant proportion of tumours, as compared to the frequent mutation and/or silencing of genes involved in adenoma-carcinoma progression [20]. We therefore concentrated our analysis on 8p, identified a region of metastasis-specific loss, and screened genes within this region for changes at the DNA, mRNA and/or protein level associated with metastasis.

## Methods

### Samples

48 sporadic CRC patients undergoing surgery at Wakefield Gastroenterology Centre for primary colon and/or secondary liver tumour resection were included, along with 20 patients with sporadic CRC and no liver metastases (follow-up 2.5–8.5 years, Mean 5.1 +/- 1.9). Matched primary colon tumour and liver metastasis samples were available for 11 patients. Informed, written consent was obtained from each patient. The Central Regional Ethics Committee approved the study (CEN/05/02/004), which complied with the Helsinki Declaration for human research. Immediately post-surgery tumour samples were dissected macroscopically to remove non-tumour tissue, snap-frozen and stored at -80°C. Blood samples were obtained for all patients.

### Nucleic acid extraction

Tumour DNA and RNA were extracted with Qiagen (Valencia, CA, USA) DNA Purification kit and Trizol reagent (Invitrogen Corp, Carlsbad, CA USA) respectively. Blood DNA was extracted using the Qiagen DNA Blood kit.

### Microsatellite markers and PCR

35 microsatellite markers, spanning 8p21-22 and part of 8q (D8S277, D8S1819, D8S351, D8S 721, D8S542, D8S520, D8S1759, D8S552, D8S1754, D8S511, D8S1827, D8S1731, D8S254, D8S261, D8S258, LPL, D8S136, D8S1786, D8S1752, D8S1734, D8S1181, D8S360, NEFL, D8S1725, D8S1739, D8S1048, D8S1809, D8S283, D8S513, D8S505, D8S325, D8S1821, D8S1745, D8S1773, D8S1833) were used. PCR used: 20 ng DNA 50 pmol each primer, 200 μM dNTPs, 1.5 mM MgCl_2_, and 0.15 units FastStart Taq (Roche Applied Science, Indianapolis, IN, US) in 50 μl volume. Cycling conditions were: 1 cycle 95°C 10 min, 30 cycles 95°C 30s 55 or 60°C 30s 72°C 30s, and 1 cycle 72°C 8 min.

### Loss of heterozygosity

As previously [21,22]. Briefly, 5 μl PCR product was denatured prior to electrophoresis and DNA visualized by silver staining. Scoring was carried out independently by 2 scientists, and a 3^rd ^scientist independently reviewed all results

### cDNA synthesis and semi-quantitative real-time PCR

500 ng of RNA was reverse transcribed using random hexamers and Superscript III (Invitrogen) as per the manufacturer. To identify a robust internal control an ABI Human Endogenous Control Plate was run against 4 paired normal colon (CN) and colon tumour (CT) samples. GAPDH (glyceraldehyde-3-phosphate dehydrogenase), acidic ribosomal protein and 18s were selected for further validation in CN, CT and liver metastases (LM). All 3 showed minimal variation between and among tissues (data not shown). TaqMan quantitative real-time PCR was performed using ABI (Applied Biosystems Foster City, CA, USA) reagents and assay on demands (Additional file 1) as per the manufacturer. Amplification efficiency and primer interference were checked using standard curves. Samples were run and analysed in triplicate on an ABI 7300 or 7700. Test gene expression was normalised to 18s (dCt). Fold change (FC) of CT or LM gene expression was calculated relative to matched normal using mean dCt values and FC = 2^-ddCt^. KRT8 was used as an epithelial cell-specific marker [23].

### Mutational analysis

PCR used: 10 ng DNA, 400 μM primer (Additional file 2), 200 μM dNTPs, 2.0 mM MgCl_2_, and 0.8 units FastStart Taq (Roche) in 30 μl, and cycling conditions: 1 cycle 95°C 4 min, and 38 cycles of 94°C 30s annealing (supplementary information) 20s 72°C 60s. DR5 (NM_003842) was amplified from cDNA as 2 over-lapping amplicons. An aliquot of each PCR product was checked before clean-up (Qiagen PCR purification columns) by agarose gel electrophoresis, sequencing was performed in both directions and anomalies verified by repeat analysis. Restriction analyses of DR4 were as previously described [24,25].

### Immunohistochemistry (IHC)

Immunohistochemistry was undertaken on formalin-fixed paraffin-embedded (FFPE) archival material. Mouse or rabbit Vectastain ABC Peroxidase Kit and Vecta Red Peroxidase substrate kits (Vector Laboratories, Burlingame, CA, USA) were used according to the manufacturer and sections counterstained with haemotoxylin. DR5 (Imgenex Corp, San Diego, CA, USA clone 45B872.1) and PDLIM2 [26] antibodies were incubated for 1 h at 37°C and used at 1:1000 and 1:500 respectively.

### Western blot

Frozen cryosections were extracted in cell lysis buffer. Tumour tissue contained >95% pure tumour as determined by haematoxylin and eosin staining of every fifth section. Samples were resolved by SDS-PAGE, transferred to nitrocellulose membrane and probed with antibodies against DR5 (1:10 000 Imgenex, clone 45B872.1), GAPDH (1:20 000 Imgenex, clone IMG-5019A-1) or ADAMDEC1 (1:600 Abnova, Taipei City, Taiwan, Clone 64C). Signals were developed using SuperSignal West Femto Maximum Sensitivity Substrate (Pierce Biotechnology, Rockford, IL, USA).

### Statistical analyses

Statistical analysis of polymorphism frequency and haplotype (LD) data involved the use of contingency tables. The strength and probability of association were measured using the R-squared statistic and chi-squared distribution respectively. Comparison of mean Ct values in real-time gene expression analyses was assessed using the independent samples T-test. An alpha level of 0.05 was set as the significance threshold.

## Results

### Identification of metastasis-specific LOH at 8p21-22

**Figure 1:**
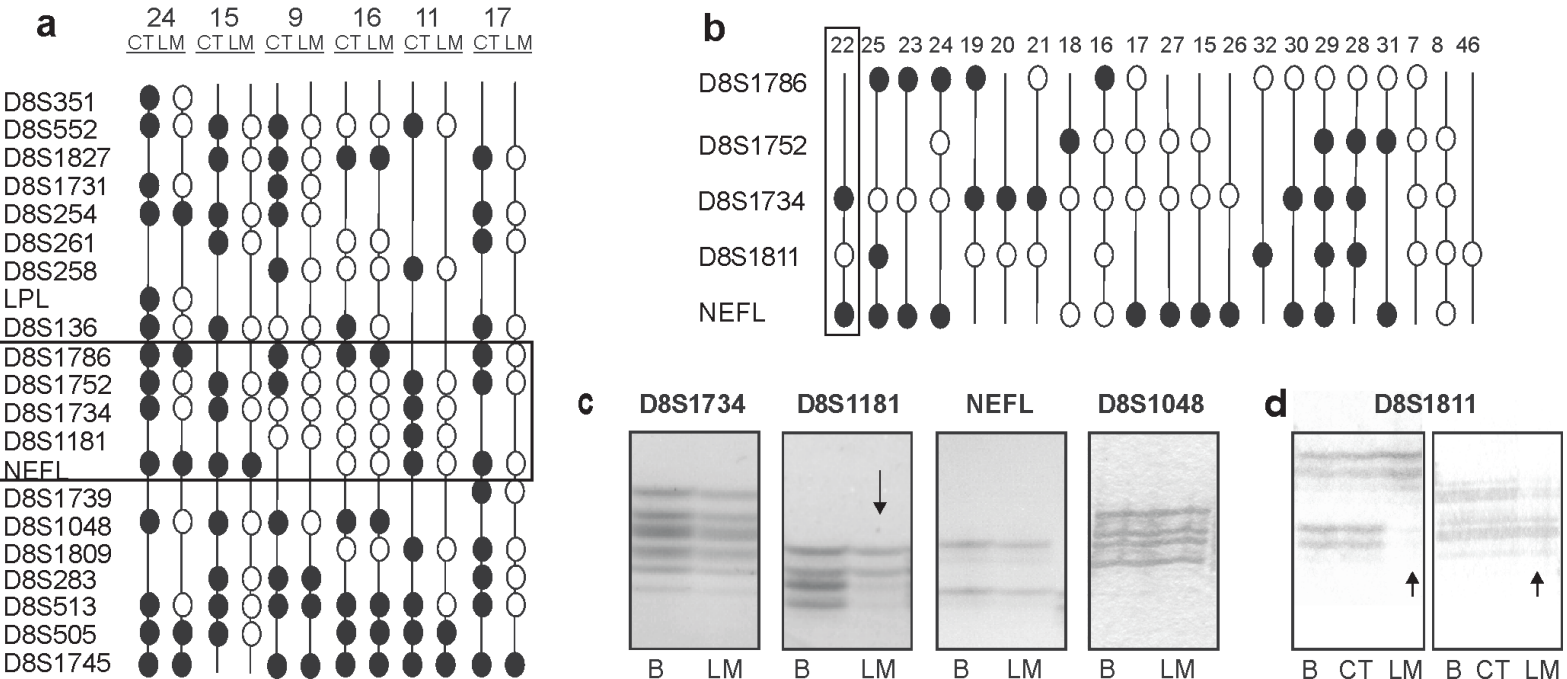
**Minimal region of LOH in CRC liver metastases**. **a**. 21 markers between D8S351-D8S1745 on chromosome 8 in matched colon tumour (CT) and liver metastases (LM) for 6 patients. Markers are ordered and oriented with telomeric end top centromeric end bottom. LOH (open circle), retention (closed circle) and Non-informative (line) are indicated. The boxed region indicates markers selected for analysis of larger series of LM samples results shown in Figure 1b. **b**. Selected markers D8S1786-NEFL on 8p21 in LM for 21 patients. Markers are ordered and oriented with telomeric end top centromeric end bottom. LOH (open circle), retention (closed circle) and Non-informative (line) are indicated. The boxed region highlights the minimal region of loss identified in patient #22. **c**. Loss of microsatellite marker D8S1181 (arrow) defining the minimal region of loss in patient #22. Retention of markers D8S1734, NEFL and D8S1048 and loss of D8S1181 in tumour (LM liver metastases) compared to normal (B, blood) are shown.**d**. Metastasis Specific Loss at D8S1181. Gel images illustrating loss (arrow) of D8S1811 in liver metastases (LM) but not matched normal (B, blood) or colon tumour (CT) in 2 patients.

LOH analysis of the entire length of chromosome 8p in matched CT (colon tumour) and LM (liver metastases) identified loss in 8/11 cases; of these, 6/8 showed regions of metastasis-specific LOH (MSL) (Figure 1a). One common region of MSL (NEFL (neurofilament protein, light polypeptide) to D8S1786) was selected for analysis in a further 36 LMs. LOH analysis of 8p21-22 in LMs revealed loss of at least one informative marker in 69% of cases (33/48) compared to matched blood. Of these 33, 15 showed loss at every informative marker analysed, suggesting loss of the entire arm of 8p (45%). 18 LMs showed discreet regions of loss (Figure 1b), and samples from one patient (#22) allowed identification of a minimal region of loss, between markers D8S1734-NEFL (2019 kb) (Figure 1c).

### Mutational analysis of candidate genes

**Figure 2:**
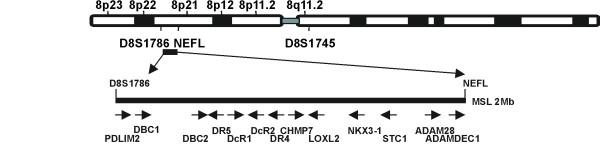
**Schematic representation of human chromosome 8p showing the minimal region of metastasis-specific loss (MSL)**. The position of microsatellite markers and the relative location and orientation of the 13 genes investigated are shown. A total of 25 protein-coding genes are located in this region (see additional information).

Examination of the published DNA sequence [27] revealed that the minimal region of MSL harboured the tumour necrosis factor-related apoptosis-inducing ligand (TRAIL) Receptor (TRAILR) gene cluster and a number of other candidates (Figure 2). TRAILR DR5 has been implicated in metastasis [19,28]. Another strong candidate, PDLIM2, is reported to promote cell attachment and migration and suppress anchorage-independent growth [26] and is involved in inactivation of NF-kappaB [29]. Therefore, we targeted TRAILRs DR5 and DR4, and PDLIM2 for mutational analyses. Of the 48 LM DNA samples used for LOH analysis, 44 had sufficient sample for mutational analyses and RNA was available for 34/44.

#### Germline termination mutation in DR5

**Figure 3:**
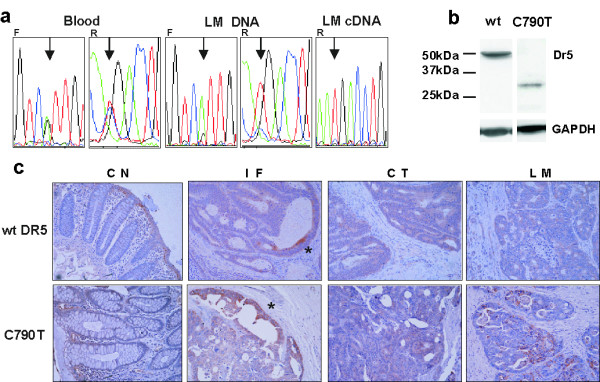
**DNA, Western and Immunohistochemical analysis of the TRAIL DR5 C790T mutation**. **a **Chromotagrams showing DR5 C790T in blood, and liver metastases DNA and cDNA. F & R indicate forward and reverse primers respectively, the arrow indicates C790T. **b**. Western Blot of DR5 for both a wild-type DR5 and the C790T LM. **c**. Immunohistochemistry for DR5 in matched colon normal (CN), the invasive front (*) of the primary colon tumour (IF), the central region of the primary tumour (CT) and liver metastasis (LM) from GAPDH is shown as a loading control. **c **both wild-type DR5 and C790T individuals.

As no particular hot-spots for mutation had previously been identified in DR5 we decided to screen the entire protein-coding region by sequencing 2 over-lapping amplicons from cDNA. Although this technique would miss any intronic splice site mutations we reasoned that it allowed a comprehensive screen for any coding mutations and that at least some splice variants might be identified as alternative amplicons. This analysis revealed known polymorphisms T95C, C200T and C572T (Table 1). A C790T transition (numbering [30]) in exon 7 was identified in one patient. C790T was predicted to introduce a premature stop codon (CGA to TGA, aa264) resulting in a truncated DR5 protein (28 kDa vs 47 kDa as primary amino acid sequence) devoid of the death domain. Sequencing of blood and LM revealed the patient as a germline heterozygote (Figure 3a) and suggested loss of the wild type allele in the LM. LM tissue was available for protein extraction for the C790T individual. This detected a protein band of approximately 28 kDa which corresponds to the predicted size of the truncated DR5 and failed to detect a band corresponding to the wild-type protein, further suggesting the possibility of loss of the wild-type allele in the LM for this patient. (Figure 3b). Matched CN, CT and LM archival samples were available for immunohistochemistry. More than 95% of cancer cells in the wild-type CT and LM and in the C790T CT were positive for DR5. However, approximately only 50% of cells were positive for DR5 in the C790T LM by immunostaining (Figure 3c). The highly DR5 positive cells were concentrated at the luminal surface in CN and at the invasive front of the CT tissues as previously reported [31]. The DR5 positive cells in the C790T LM, some of which had a high level of expression, were morphologically distinct and poorly differentiated (Figure 3c).

**Table 1:** Frequency of Polymorphisms in Liver Metastases Samples

**Polymorphism**	**Frequency**
DR4 G422A Exon1	35/44
DR4 C626G Exon 2	35/44
DR4 A683C Exon 5	13/44
DR4 A+12Ex5G Intronic	27/44
DR4 A1322G DD Exon 9	7/44
DR5 T95C Exon 1	22/34
DR5 C200T Exon 2	3/34
DR5 C572T Exon 5	6/34
PDLIM2 C1281T Exon 9	3/44

#### Novel DR4 polymorphism in metastasising CRC

We targeted the cysteine-rich extracellular ligand binding domain (exons 3, 4 and 5), and intracellular death domain (exon 9). DNA sequencing and confirmatory restriction analysis detected known polymorphisms G422A, C626G, and A683 (Table 1).

The G allele of SNP (single nucleotide polymorphism) rs4872077 was significantly more frequent in individuals with LM (27/44, 6 homozygous G, 21 heterozygotes) than those without (6/20, all heterozygous) p = 0.02. rs4872077 was in linkage disequilibrium with two DR4 polymorphisms G422A and C626G (r^2 ^= 0.50 and 0.48 respectively, p = 0.001) previously shown to co-segregate [24]; in our study the linkage disequilibrium between G422A and C626G was r^2 ^= 0.914, p < 3 × 10^-17^.

#### Novel polymorphism in PDLIM2

We targeted the PDZ and LIM domains which have roles in the assembly of protein complexes (PDZ, [32]) and act as protein binding interfaces found in transcription factors, kinases and scaffolding proteins (LIM, [33]). The two domains span several exons and were amplified independently from cDNA. A novel, heterozygote C to T transition at nt1281 (NM_021630) was detected (3/34). All three were heterozygous for C1281T in germline DNA (data not shown).

### Gene expression analysis for CRC metastasis

Mutation is one mechanism of gene disruption. To provide a general screen at the transcriptional level we extended our search to a semi-quantitative real-time PCR analysis of DR5, DR4, PDLIM2 and a further 10 potential candidates (Figure 2). The 2 Mb region of MSL harbours 25 protein-coding genes ([27] and Additional file 3). At the time of study we picked the next 10 best candidates based on a literature search and sequence alignments (DNA & protein) to reveal any pertinent homologies or potential functions. These were: the TRAIL decoy receptors DcR1 and DcR2; DBC1 which is homozygously deleted in breast cancers [34], and has a role in apoptosis [35,36]; DBC2 a RhoGTPase and putative breast tumour suppressor gene [34] implicated in apoptosis, cell cycle control, cytoskeleton and membrane trafficking [37,38] and down-regulation of Cyclin D1 [39]; STC1 which is induced by BRCA1 and VEGF and up-regulation is reported in a number of cancers [4,40]; LOXL2 which is differentially expressed in various tumours [41-43] and interacts with SNAIL [44,45]; CHMP7 which may function in endosomal sorting [46]; NKX3.1 a prostate tumour suppressor [47,48], and two members of the ADAM (a disintegrin and metalloproteinase) family (ADAM28 and ADAMDEC1) whose members play a role in carcinoma progression[49].

**Figure 4:**
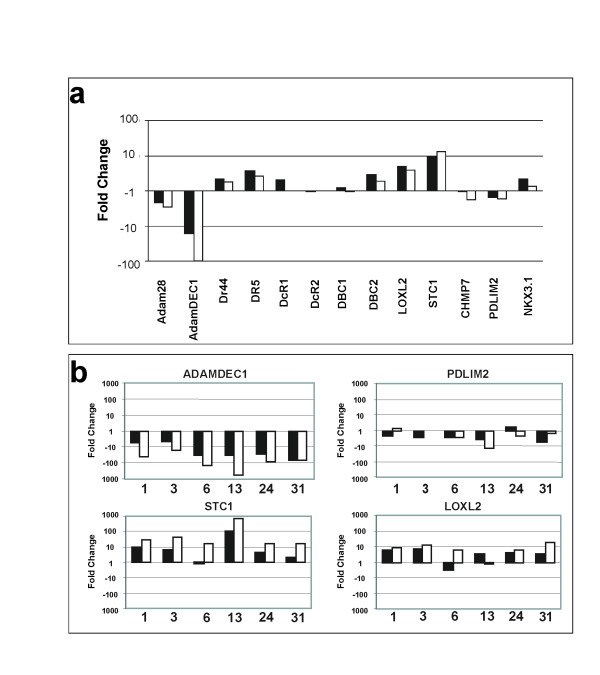
**mRNA gene expression analyses for 13 genes**. **a**. Fold change gene expression in colon tumour (black) and liver metastases (white) relative to colon normal. Gene expression was expressed as the change in Ct of the gene of interest compared to the 18s control (dCt) and relative expression calculated using the comparative C_T _method with fold change (2^-ddCt^). Fold change is shown on a logarithmic scale. **b**. Fold change gene expression of colorectal tumours (black) and liver metastases (white) relative to the matched colon normal. Fold change is presented on a logarithmic scale and was calculated as above.

mRNA levels were investigated in 34 LM samples and 14 matching CT samples. 6 colon normal (CN) samples with matching LM and CT (as above) were also analysed. Mean expression data for the 13 genes across the 3 tissues was calculated (Additional file 4) and mean fold change expression in colon tumour and liver metastases relative to colon normal is presented in Figure 4a.

#### ADAMDEC1

**Figure 5:**
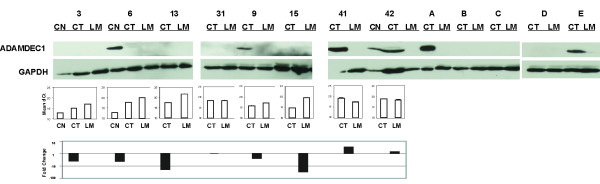
**Western analysis of ADAMDEC1**. Analysis of matched CN (colon normal), CT (colon tumour) and LM (liver metastasis) for 13 patients is shown. GAPDH was used as a loading control. Numbers refer to patients IDs and allow cross-referencing to LOH and gene expression data where appropriate. Letters correspond to additional samples for whom mRNA expression and LOH was not undertaken. Where available mRNA expression is shown as mean dCt (Y-axis) underneath the corresponding sample and Fold Change for LM relative to CT is presented.

A striking decrease in ADAMDEC1 mRNA expression was observed in CT compared with paired CN; expression decreased further in matched LM (Figure 4b). Data from the NCBI Geo profile [50] supports this observation. Varying levels of ADAMDEC1 mRNA have been detected in normal human colon (GDS2062, GDS829, GDS1096, GDS559, and original publication [51]). Two studies (GDS2062 and GDS829) included normal colon and colon adenocarcinoma cell lines, SW480 or CaCo2, and expression was detected in normal tissue with very low or undetectable levels in the cell lines. ADAMDEC1 protein expression was detected in 12/20 normal adjacent colon samples by Western Blot (data not shown). Western analysis of ADAMDEC1 expression in 13 matched CT and LM samples (3 with matching CN) detected protein in 2/3CN, 5/13 CT and 0/13 LM samples (representative western shown in Figure 5).

Six patients had a family history of CRC (first and/or second degree relatives). ADAMDEC1 mRNA expression was decreased to a lesser extent in the LM samples of these patients relative to those with no family history (4-fold difference in expression, T-test p = 0.02).

#### PDLIM2

A decrease in mRNA expression was observed for PDLIM2 in 5/6 CT samples compared with matched CN (Figure 4b). Immunohistochemistry did not reveal any difference in the level or location of protein expression between matched CT and LM (Additional file 5).

#### STC1 & LOXL2

We observed increased mRNA expression for STC1 and LOXL2 in tumour tissue (Figure 4b), consistent with previous reports [4,40,41,43].

#### DcR1

Relationships between gene expression (all 13 genes) and clinical outcome were investigated using clinical parameters of survival: development of extra hepatic disease post-LM resection; 3 year survival; histological grade of the CT, and presence of metachronous or synchronous metastases. A 2.5 fold lower mRNA expression of DcR1 in LM samples was associated with an increased likelihood of extra hepatic disease at 12 months post liver tumour resection. (T-test p = 0.005, n = 30. Data not shown). In CT samples there was a trend towards this association (T-test p = 0.051, n = 14, fold change of 2.4).

## Discussion

Disruption of 8p is common in many cancers and could simply indicate the relative instability of this region such that disruption is a consequence of disease rather than playing a causative role in tumour progression. In CRC 8p loss has been implicated in later stage disease and metastasis [13-15] and this study again highlights 8p identifying a 2 Mb region of metastasis-specific loss at 8p21-22. In addition, there is compelling evidence for the role of several 8p genes in carcinogenesis, including a number studied here, such as the prostate tumour suppressor NKX3.1, TRAILR DR5, DBC2 and STC1. This tends to argue against 8p disruption being primarily a consequence of tumourigenesis.

In this study the most promising candidate at the mRNA level was ADAMDEC1. We observed decreased mRNA expression in CT and LM compared to CN (Figure 4b). No detectable protein was expressed in 13 LM, and in 3/13 matched CT samples ADAMDEC1 was detected which adds weight to the possibility that loss of expression is progressive through tumourigenesis and may play a role in metastasis. (Figure 5). ADAM family members are involved in cancer progression[49] adding further support to the possibility that this unique family member also has a role in tumourigenesis.

We also identified a germline mutation of DR5 which may provide additional insights into the function of this TRAILR. C790T truncates DR5 (Figure 3b) resulting in a protein devoid of the death domain and a potential functional similarity to DcR2. Several studies have also identified truncating mutations of DR5 located prior to the death domain [52-55]. One (another germline mutation) showed loss of the growth suppressive function of wild-type DR5 in HNSCC (head and neck squamous cell carcinoma), ovarian and CRC cell lines [53]. Despite the diagnosis of synchronous liver metastases the C790T patient did not develop extra-hepatic metastases, responded well to hepatic chemotherapy (5-flurouracil (5-FU)) and SIRT (selective internal radiation) and had > 5-year survival post LM diagnosis. Inducible loss of DR5 protein expression promotes the growth of colon tumours in mice and confers resistance to 5-FU, without causing resistance to TRAIL-induced apoptosis [56]. This is in contrast to the clinical observations for the C790T patient (slow tumour growth and p53-responsiveness to 5FU and SIRT).

The heterogeneous or "patchy" C790T DR5 immunostaining and lack of a wild-type DR5 in C790T LM by western analysis suggests complete loss of the wild-type DR5 in C790T LM. The clinical data indicate that the C790T DR5 may have a dominant negative effect, similar to DcR2, including retention of the ability to respond to p53-dependent therapy, and prevention of controlled proliferation, in contrast to null DR5 experimental models [56].

In light of the wealth of evidence implicating 8p in CRC progression it is perhaps surprising that no strong candidate tumour and/or metastatic suppressor has been identified. Other possibilities are that the 'candidate(s)' could be a microRNA or other non-coding RNA or that haploinsufficiency (rather than gene 'knock-out') is sufficient for tumourigenesis. Alternatively, a single gene/RNA may not be the main 'effector' but rather it could be a combinatorial effect whereby a number of genes are involved and perturbation of them all, or a subset thereof, results in tumour progression.

The 2 Mb region in this study certainly appears to be a hot-spot for genes involved in carcinogenesis, and contains 3 gene clusters, TRAILR, ADAM and NKX3.1/NKX2.6, each encoding members with a role in tumour progression. There is evidence for the clustering of co-expressed genes in eukaryotes [57], as well as increasing recognition that dynamic chromosomal architecture and genomic repositioning play an important role in gene regulation [58]. An indication that clustering is of functional importance at 8p21-22 is suggested by the observation of co-regulation at the mRNA transcriptional level and co-methylation patterns for the TRAILR pairs DcR1 and DcR2 and DR4 and DR5 in neuroblastoma cell lines [59]. We performed Pearsons' correlation analysis on the gene expression data for the 13 candidate genes in both CT and LM. This revealed potential transcriptional relationships between a number of genes including DR4 and DR5 (Additional file 6) which adds support to the possibility that the clustering of 'tumour' genes within this region is of functional significance.

In addition, this possibility is further supported by recent evidence demonstrating that the nuclear protein SATB1 acts as a 'genomic organiser' involved in the epigenetic remodelling of chromatin to facilitate upregulation of metastasis-associated genes and down-regulation of tumour suppressors [60]. Although the genes investigated by Han et al were not clustered, 8p21-22 may be a key candidate target of such regulation, the clustering providing a further mechanism for co-ordinated control.

Metastasis might be viewed as a complex disorder in which genetic and environmental factors interact, subtle modulations of cellular activity being required to facilitate survival. We propose that 8p21-22 may not contain a CRC metasatasis suppressor(s) and that the clustering of a large number of genes in one region under co-ordinated control bears closer resemblance to a complex disease, whereby the overall combined profile of multiple genes contributes to the phenotype.

## Conclusion

This study identified a metastatic susceptibility locus within a 2 Mb region of 8p21-22, which appears to be a "hot-spot" for genes with a role in carcinoma development and revealed ADAMDEC1 as a potential tumour suppressor. We suggest that the rich nature of this region for genes with a role in tumour development is of pathological significance such that the genes may form a cluster disruption of which favours CRC tumour progression. The possibility of relationships between the genes is supported by the presence of several gene clusters, our observation of potential transcriptional associations, and van Noesels et al observation of transcriptional co-regulation and co-methylation of the DR4/DR5 and DcR1/DcR2 TRAILR pairs [59]. As understanding of the role of genomic architecture increases it may become essential to consider neighbouring genes (in genomic and/or nuclear space) to fully understand both gene function and carcinoma progression.

## Abbreviations

CRC: colorectal cancer; CN: colon normal; CT: colon primary tumour; LM: liver metastasis; LOH: loss of heterozygosity; FFPE: formalin-fixed paraffin-embedded; IHC: immunohistochemistry; MSL: metastasis specific loss.

## Competing interests

The authors declare that they have no competing interests.

## Authors' contributions

DPM–C carried out the semi-quantitative real-time PCR, contributed to the mutation analyses and study design, and drafted the manuscript. KAH participated in the IHC, study design and manuscript preparation. AW and HjS performed the IHC. TW carried out the mutation analyses. RO'C was involved in the IHC and critical reading of the manuscript. DAH and RAL performed the statistical analyses. JAR participated in IHC, study design and manuscript preparation. RSS provided clinical expertise, clinical samples and critical reading of the manuscript. SR conceived the study, participated in its design, performed LOH analyses, and helped with manuscript preparation. All authors read and approved the final manuscript.

## Pre-publication history

The pre-publication history for this paper can be accessed here:



## Supplementary Material

Additional file 1 Assays from ABI used for real-time PCR. Click here for file

Additional file 2 Oligonucleotide primers and annealing temperatures. Click here for file

Additional file 3 25 protein-coding genes encoded by 2 Mb region of MSL. Click here for file

Additional file 4 Gene expression data (Mean dCt and SEM) for all genes investigated. Click here for file

Additional file 5 Immunohistochemistry of PDLIM2. Click here for file

Additional file 6 Gene:gene mRNA expression correlations in CT and matched LM. Click here for file
