# Supplementary material for: Metastatic susceptibility locus, an 8p hot-spot for tumour progression disrupted in colorectal liver metastases: 13 candidate genes examined at the DNA, mRNA and protein level
Source: BMC Cancer. 2008 Jul 1;8:187. doi: 10.1186/1471-2407-8-187 (PMC2488356; doi:10.1186/1471-2407-8-187)
Supplement: Additional file 1 — Assays from ABI used for real-time PCR. [file 1471-2407-8-187-S1.doc]

**1) Assays from ABI** used for real-time PCR.

| **Gene** | **Assay used (ABI)** |
| --- | --- |
| DR4/TNFRSF10A | Hs00269491_m1 |
| DR5/TNFRSF10B | Hs0036672_m1 |
| DcR1/TNFRSF10C | Hs00427795_g1 |
| DcR2/TNFRSF10D | Hs00388742_m1 |
| DBC1/KIA1967 | Hs00368356_m1 |
| DBC2/RhoBTB2 | Hs00248529_m1 |
| CHMP7 | Hs00292844_m1 |
| LOXL2 | Hs00158757_m1 |
| ADAM28 | Hs00248020_m1 |
| ADAMDEC1 | Hs00205545_m1 |
| PDLIM2 | Hs00222154_m1 |
| NKX3.1 | Hs00171834_m1 |
| STC1 | Hs00174970_m1 |
| GAPDH | 4326317E (Vic/MGB, primer limited) |
| Human RPLPO | 4326314E (Vic/MGB, primer limited) |
| 18s | 4319413E (Vic/MGB, primer limited) |
