# Supplementary material for: Metastatic susceptibility locus, an 8p hot-spot for tumour progression disrupted in colorectal liver metastases: 13 candidate genes examined at the DNA, mRNA and protein level
Source: BMC Cancer. 2008 Jul 1;8:187. doi: 10.1186/1471-2407-8-187 (PMC2488356; doi:10.1186/1471-2407-8-187)
Supplement: Additional file 2 — Oligonucleotide primers and annealing temperatures. [file 1471-2407-8-187-S2.doc]

**2**) Oligonucleotide primers and annealing temperatures.

| Primer | Forward | Reverse | Annealing ºC |
| --- | --- | --- | --- |
| DR4 Exon 3 | 5’ ATCCTCTGGGAACTCTGTGG 3’ | 5’ TACCACTCCCACCTCACTGC 3’ | 58 |
| DR4 Exon 4 | 5’ AAGGTCAAGGGACACGTCAAG 3’ | 5’ GCTTCTGTGGTTTCTTTGAGG 3’ | 58 |
| DR4 Exon 5 | 5’ ATCCCACCTGGCCAGCTTTCCA 3’ | 5’ AGACAGGAGTCTCGGGCTGCT 3’ | 56 |
| DR4 death domain exon10 | 5’ CTCTGATGCTGTTCTTTGAC 3’ | 5’ TCACTCCAAGGACACGGCAG 3’ | 56 |
| DR5 coding region ‘A’ | 5’ GTTCCCTACCGCCATGGAACA 3’ | 5’ GATGATGCCTGAGAGAGAAC 3’ | 60 |
| DR5 coding region ‘B’ | 5’ GAATCAGGTACAAAGCACAG 3’ | 5’ GGTAAACCAGGGAAGGTCTGA 3’ | 60 |
| DR5 exon 7 | 5’ TCGGCTTTTTGCCTTCCCAATGTC 3’ | 5’ GAAACAAAATGATCTGTCCCCCACT 3’ | 65 |
| PDLIM PDZ domain | 5’ GGAGTCCACTGACCGGCTCAAA 3’ | 5’ CTTCCAAGGAGCTGTCCCCATT 3’ | 58 |
| PDLIM LIM domain | 5’ GAGGTGGCACGCCAGCCTTCTT 3’ | 5’ CAGTGAGGCAGGCTGAGGGCAT 3’ | 72 |
| PDLIM exon 9 | 5’ CCTTACCTGGTGCCACTGTCA 3’ | 5’ CTAGAGTCCCCGACTGGGTA 3’ | 60 |
