# Supplementary material for: Metastatic susceptibility locus, an 8p hot-spot for tumour progression disrupted in colorectal liver metastases: 13 candidate genes examined at the DNA, mRNA and protein level
Source: BMC Cancer. 2008 Jul 1;8:187. doi: 10.1186/1471-2407-8-187 (PMC2488356; doi:10.1186/1471-2407-8-187)
Supplement: Additional file 3 — 25 protein-coding genes encoded by 2 Mb region of MSL. [file 1471-2407-8-187-S3.doc]

| **Gene** | **Protein** | **Ensembl ID** |
| --- | --- | --- |
| **PDLIM2/Mystique2** | **PDZ & LIM domain protein** | **ENSG00000120913** |
| C8orf58 | unknown | ENSG00000157927 |
| **DBC1 –** | **deleted in breast cancer 1** | **ENSG00000158941** |
| BIN3 | Bridging integrator 3 | ENSG00000147439 |
| EGR3 | early growth response protein 3 | ENSG00000179388 |
| PEBPL_Human | PEBP family precursor | ENSG00000134020 |
| **DBC2/RHOBTB2** | **deleted in breast cancer 2, a RhoGTPase** | **ENSG00000008853** |
| **TRAILR DR5** | **TRAIL receptor** | **ENSG00000120889** |
| **TRAILR DcR1** | **TRAIL receptor** | **ENSG00000173535** |
| **TRAILR DcR2** | **TRAIL receptor** | **ENSG00000173530** |
| **TRAILR DR4** | **TRAIL receptor** | **ENSG00000104689** |
| **CHMP7** | **CHMP family member . charged multivesicular body protein 7 /chromatin-modifying protein 7** | **ENSG00000147457** |
| R3HCC1 | R3H domain & coiled-coil domain containing protein | ENSG00000104679 |
| **LOXL2** | **Lysyl-oxidade-like 2** | **ENSG00000134013** |
| ENTPD4/NTPDase4 | Ectonucleosie triphosphate diphosphohydrolase 4 | ENSG00000197217 |
| SLC25A37 | Mitoferrin-1, Mitochondrial solute carrier protein | ENSG00000147454 |
| Q9P1G9_Human | unknown | ENSG00000180959 |
| Q16458_Human | unknown | ENSG00000215298 |
| Q71JB2_Human | unknown | ENSG00000205612 |
| **NKX3.1** | **NK homeobox family 3. Prostate tumour suppressor** | **ENSG00000167034** |
| NKX2.6 | Cardiac specific homeobox 2 | ENSG00000180053 |
| **STC1** | **Stanniocalcin 2** | **ENSG00000159167** |
| **ADAM28** | **A disintegrin and metalloproteinase family** | **ENSG00000042980** |
| **ADAMDEC1** | **A disintegrin and metalloproteinase-domain-like decysin** | **ENSG00000134028** |
| ADAM7 | A disintegrin and metalloproteinase family | ENSG00000069206 |

Table showing the 25 protein-coding regions encoded between D8S1786 and NEFL ([www.ensembl.org](http://www.ensembl.org/)) in the region of metastatic specific loss identified on chromosome 8p. The 13 candidate genes included in the study are highlighted in bold.

ADAM7 was selected for real-time analysis, but the ABI gene expression assay failed to detect expression in any tissue tested.
