# Supplementary material for: Metastatic susceptibility locus, an 8p hot-spot for tumour progression disrupted in colorectal liver metastases: 13 candidate genes examined at the DNA, mRNA and protein level
Source: BMC Cancer. 2008 Jul 1;8:187. doi: 10.1186/1471-2407-8-187 (PMC2488356; doi:10.1186/1471-2407-8-187)
Supplement: Additional file 4 — Gene expression data (Mean dCt and SEM) for all genes investigated. [file 1471-2407-8-187-S4.doc]

| **Gene** | **Colon Normal** | | **Colon Tumour** | | **Liver Metastases** | |
| --- | --- | --- | --- | --- | --- | --- |
|  | **Mean dCt** | **SEM** | **Mean dCt** | **SEM** | **Mean dCt** | **SEM** |
| **Adam28** | 18.6 | 0.19 | 19.7 | 0.24 | 20.1 | 0.26 |
| **ADAMDEC1** | 12.5 | 0.13 | 16.6 | 0.46 | 18.6 | 0.33 |
| **DR4** | 16.4 | 0.12 | 15.2 | 0.16 | 15.5 | 0.17 |
| **DR5** | 19.5 | 0.12 | 17.6 | 0.18 | 18.0 | 0.20 |
| **DcR1** | 21.0 | 0.08 | 19.9 | 0.40 | 21 | 0.24 |
| **DcR2** | 19.6 | 0.24 | 19.7 | 0.19 | 19.6 | 0.19 |
| **DBC1** | 16.6 | 0.08 | 16.3 | 0.14 | 16.7 | 0.14 |
| **DBC2** | 20.5 | 0.30 | 19.0 | 0.25 | 19.6 | 0.22 |
| **LOXL2** | 16.4 | 0.10 | 14.1 | 0.37 | 14.4 | 0.26 |
| **STC1** | 22.8 | 0.32 | 19.6 | 0.45 | 19.1 | 0.31 |
| **CHMP7** | 16.4 | 0.08 | 16.5 | 0.19 | 17.2 | 0.13 |
| **PDLIM2** | 12.5 | 0.24 | 13.1 | 0.35 | 13.2 | 0.21 |
| **NKX3.1** | 22.8 | 0.34 | 21.6 | 0.38 | 22.3 | 0.26 |
| **KRT8** | 10.4 | 0.16 | 10.7 | 0.24 | 10.5 | 0.23 |

Table presenting gene expression data for the 13 candidate genes and control gene KRT8 in colon normal, colon tumour and liver metastases tissues. The Mean dCt value and standard error of the mean (SEM) are shown. Gene expression is expressed as the change in Ct of the gene of interest compared to the 18s control (dCt). The SEM is presented to facilitate visual interpretation of whether there is a statistically significant difference in gene expression between tissue types for each gene (Altman DG & Bland, J.M. Standard deviations and standard errors. British Medical Journal 2005, **331**: 903).
