# Supplementary material for: Metastatic susceptibility locus, an 8p hot-spot for tumour progression disrupted in colorectal liver metastases: 13 candidate genes examined at the DNA, mRNA and protein level
Source: BMC Cancer. 2008 Jul 1;8:187. doi: 10.1186/1471-2407-8-187 (PMC2488356; doi:10.1186/1471-2407-8-187)
Supplement: Additional file 5 — Immunohistochemistry of PDLIM2. [file 1471-2407-8-187-S5.pdf]

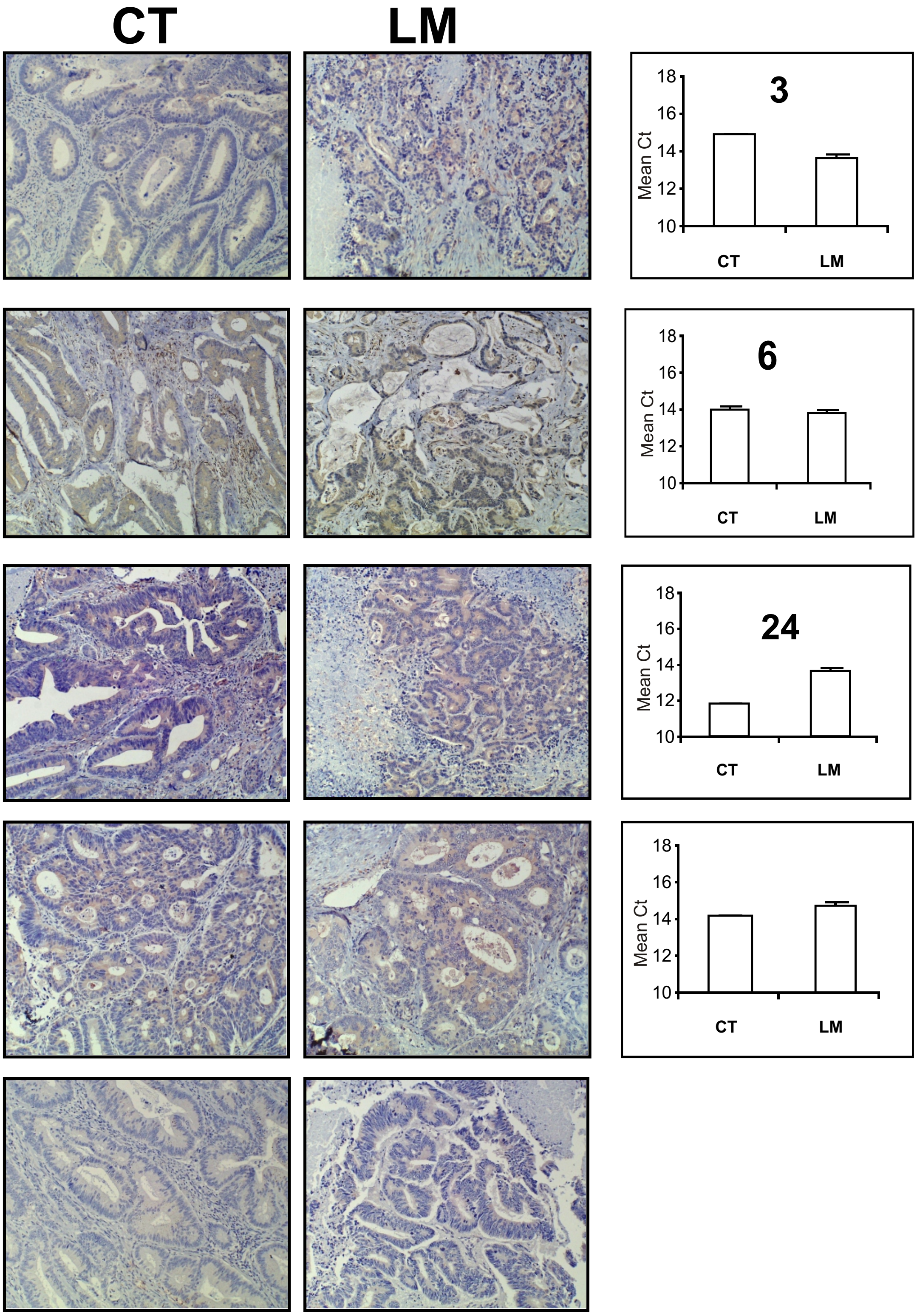

Immunohistochemical staining for PDLIM2 in matched colon tumour (CT) and liver metastasis (LM) tissue.

Where available mean dCt values (with SEM) for mRNA expression are given. Numbers 3, 6 and 24 correspond to patients in Figure 4b. Protein expression was homogenously expressed throughout the tumour, restricted to epithelial cells and no staining of stroma cells was observed. PDLIM2 protein levels varied from low to high between patients in CT and LM. There was no difference in the level of PDLIM2 protein observed between matched CT and LM as detected by immunohistochemistry.
