# Supplementary material for: Metastatic susceptibility locus, an 8p hot-spot for tumour progression disrupted in colorectal liver metastases: 13 candidate genes examined at the DNA, mRNA and protein level
Source: BMC Cancer. 2008 Jul 1;8:187. doi: 10.1186/1471-2407-8-187 (PMC2488356; doi:10.1186/1471-2407-8-187)
Supplement: Additional file 6 — Gene:gene mRNA expression correlations in CT and matched LM. [file 1471-2407-8-187-S6.pdf]

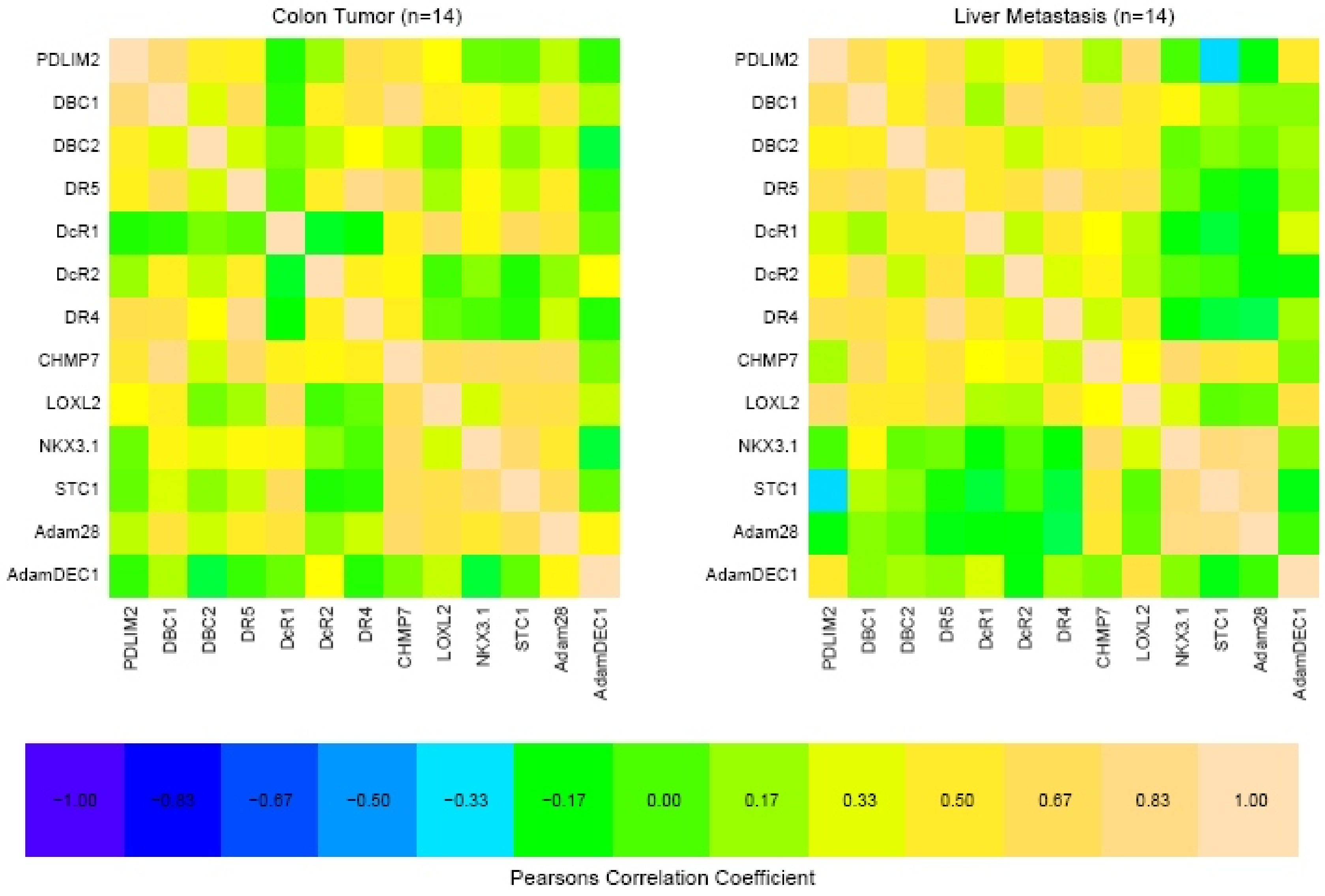

Gene-by-gene mRNA expression correlations in colon tumour and matched liver metastasis samples (n=14).

To investigate the possibility of transcriptional relationships between the 13 candidate genes and to see whether any such relationship might differ between the two tumour tissues we used Pearson's correlation analysis. Expression data (Mean dCt) for each gene in 14 matched CT and LM was interrogated. The analysis revealed potential transcriptional relationships between a number of genes in the two tissues.
